# Alpha-galactosylceramide enhances mucosal immunity to oral whole-cell cholera vaccines

**DOI:** 10.1038/s41385-019-0159-z

**Published:** 2019-04-05

**Authors:** Christopher J. H. Davitt, Stephanie Longet, Aqel Albutti, Vincenzo Aversa, Stefan Nordqvist, Becky Hackett, Craig P. McEntee, Monica Rosa, Ivan S. Coulter, Michael Lebens, Joshua Tobias, Jan Holmgren, Ed C. Lavelle

**Affiliations:** 1 Adjuvant Research Group, School of Biochemistry and Immunology, Trinity Biomedical Sciences Institute, Trinity College Dublin, Dublin 2, D02 R590 Ireland; 2 College of Applied Medical Sciences, Qassim University, Buraydah, 52571 Saudi Arabia; 3 Sublimity Therapeutics (Holdco) Ltd, DCU Alpha Innovation Campus, Old Finglas Road, Dublin, D11 KXN4 Ireland; 4 Department of Microbiology and Immunology, University of Gothenburg Vaccine Research Institute (GUVAX), University of Gothenburg, Box 435, 405 30 Gothenburg, Sweden; 5 Centre for Research on Adaptive Nanostructures and Nanodevices (CRANN) & Advanced Materials Bio-Engineering Research Centre (AMBER), Trinity College Dublin, Dublin 2, D02 PN40 Ireland

## Abstract

Cholera is a severe diarrheal disease caused by the bacterium *Vibrio cholerae* (*V. cholerae*) that results in 3–4 million cases globally with 100,000–150,000 deaths reported annually. Mostly confined to developing nations, current strategies to control the spread of cholera include the provision of safe drinking water and improved sanitation and hygiene, ideally in conjunction with oral vaccination. However, difficulties associated with the costs and logistics of these strategies have hampered their widespread implementation. Specific challenges pertaining to oral cholera vaccines (OCVs) include a lack of safe and effective adjuvants to further enhance gut immune responses, the complex and costly multicomponent vaccine manufacturing, limitations of conventional liquid formulation and the lack of an integrated delivery platform. Herein we describe the use of the orally active adjuvant α-Galactosylceramide (α-GalCer) to strongly enhance intestinal bacterium- and toxin-specific IgA responses to the OCV, Dukoral^®^ in C57BL/6 and BALB/c mice. We further demonstrate the mucosal immunogenicity of a novel multi-antigen, single-component whole-cell killed *V. cholerae* strain and the enhancement of its immunogenicity by adding α-GalCer. Finally, we report that combining these components and recombinant cholera toxin B subunit in the SmPill^®^ minisphere delivery system induced strong intestinal and systemic antigen-specific antibody responses.

## Introduction

Cholera is mostly caused by enteric infections with *Vibrio cholerae* (*V. cholerae*) of the El Tor biotype and O1 serogroup (and in rare cases *V. cholerae* El Tor of serogroup O139).^1,2^ The disease is endemic in large parts of South-East Asia and Africa and has also caused major outbreaks in many developing countries. Oral cholera vaccines (OCVs) have proved effective against endemic cholera after vaccination and have been shown to reduce cholera incidence in outbreaks by >50%.^3^

Dukoral^®^ was the first whole-cell killed (WCK) OCV to be licensed globally. It is composed of WCK bacteria of the different serotypes (Inaba and Ogawa) and biotypes (Classical and El Tor) of the O1 serogroup of *V. cholerae* together with recombinantly produced cholera toxin B subunit (CTB), thus combining bivalent protection against bacterial colonization and cholera toxin (CT). Three other licensed WCK OCVs, Orc-Vax^TM^, Shanchol^TM^, and Euvichol contain the same WCK *V. cholerae* O1 strains as Dukoral^®^ along with WCK bacteria of the O139 serotype.^4^ However, unlike Dukoral^®^ these do not contain CTB.^4^

While these vaccines have shown efficacy in clinical and field trials, global distribution and uptake, where they are most needed, is still challenging.^1^ The long term protective efficacy is moderate and their production, storage, distribution, and administration are expensive. All currently licensed OCVs require production and inactivation of individual batches of bacteria expressing the various antigenic targets under good manufacturing practice (GMP) conditions; while the CTB in Dukoral^®^ is expressed and purified from a genetically de-toxified strain of *V. cholerae*. They also require cold chain transport and storage and Dukoral^®^ requires the use of a bicarbonate buffer to neutralize stomach acidity to protect the acid-labile CTB subunit from degradation.^4^

To simplify production of OCVs, Lebens et al.^5,6^ recently developed stable Inaba and Ogawa-LPS expressing ‘Hikojima’ phenotype strains of *V. cholerae* that require only formalin inactivation, eliminating the need for different batches of *V. cholerae* to be grown and inactivated by different processes. One such El Tor Hikojima WCK OCV (Hillchol^TM^) is being evaluated and has exhibited comparable safety and immunogenicity to Shanchol^TM^ in side-by-side “non-inferiority” comparison clinical studies (ClinicalTrials.gov Identifier: NCT02823899). The utilization of adjuvants may enhance immunogenicity and facilitate longer term protection compared to currently licensed OCVs.^7^ However, to date no adjuvants have been included in licensed oral vaccines. Recently, we demonstrated that the invariant Natural Killer T (iNKT) cell activator α-Galactosylceramide (α-GalCer) potentiated mucosal immune responses against an experimental oral enterotoxigenic *Escherichia coli* (ETEC) vaccine.^8^ Delivery technologies that protect labile vaccine components from gastric acidity and enzymatic degradation can enhance immunogenicity.^9^ We reported the ability of the Single-Multiple Pill^®^ (SmPill^®^) platform to enhance intestinal and systemic antigen-specific antibody responses against a WCK ETEC oral vaccine^8^ as well as maintain vaccine formulation integrity and antigenicity following storage under non-refrigerated conditions.^10^

Here, we show that combining α-GalCer with the novel WCK *V. cholerae* strain and recombinant CTB subunit in the oral-delivery SmPill^®^ minispheres offers a promising novel approach to developing an improved WCK oral cholera vaccine with increased mucosal immunogenicity compared to currently licensed vaccines.

## Results

### α-GalCer significantly enhanced intestinal and serum antigen-specific antibody responses after oral co-administration with Dukoral^®^ in C57BL/6 and BALB/c mice

The propensity for orally administered antigens to promote oral tolerance and the tropical barrier are two hurdles that need to be overcome to implement an effective oral vaccination strategy.^9^ Mucosal adjuvants are required to enhance the immunogenicity of orally delivered antigens. However, there are currently no efficacious and safe adjuvants approved for oral administration.^11^

**Fig. 1:**
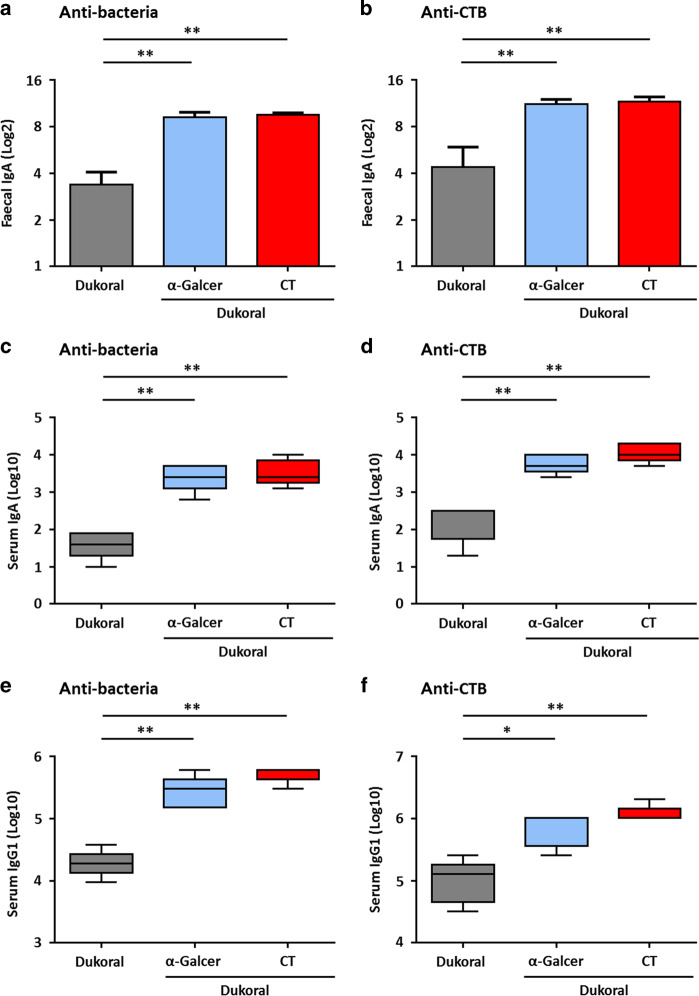
α-GalCer enhances antigen-specific faecal IgA, serum IgA and IgG1 responses after oral vaccination compared to Dukoral^®^ alone in female C57BL/6 mice. Mice were immunized orally with Dukoral^®^ either with or without α-GalCer or CT as an adjuvant. Faecal pellets and sera were collected on day 41 and 42, respectively. Bacteria-specific and CTB–specific IgA responses in faecal pellets (**a**, **b**) and serum (**c**, **d**) as well as bacteria-specific and CTB-specific IgG1 responses in serum (**e**, **f**) were measured and antibody titres were determined by end-point ELISA. Graphs present mean titres (+SEM) for five mice per experimental group. *p < 0.05, **p < 0.01

In order to evaluate the ability of α-GalCer to increase immune responses to an OCV, intestinal, and systemic antigen-specific antibody responses after oral vaccination with Dukoral^®^ alone or combined either with α-GalCer or CT were compared in female C57BL/6 mice. Administration of α-GalCer significantly increased bacteria- and CTB-specific IgA intestinal responses as measured in faecal pellet supernatants (FPS) compared to Dukoral^®^ alone and in a comparable manner to Dukoral^®^ adjuvanted with CT (Fig. 1a, b). This enhancement of intestinal antibody responses by α-GalCer was also observed systemically with both bacteria- and CTB-specific IgA titres in serum being significantly higher after administration of the Dukoral^®^ adjuvanted with α-GalCer compared to the vaccine alone and similar to the vaccine adjuvanted with CT (Fig. 1c, d). Dukoral^®^ adjuvanted with α-GalCer also generated significantly higher systemic bacteria- and CTB-specific IgG1 responses as measured in serum compared to Dukoral^®^ alone and responses were comparable to those following administration of Dukoral^®^ administered with CT (Fig. 1e, f). To address whether these responses were more broadly applicable across mouse strains and sexes, the effectiveness of α-GalCer was also addressed in male C57BL/6 mice and female BALB/c mice. While Dukoral^®^ alone induced bacteria- and CTB-specific IgA responses in FPS, α-GalCer enhanced these responses in males, although to a lesser extent than that seen in females (Suppl Fig. 1a, b). However, the increase in bacteria- and CTB-specific IgA responses induced by α-GalCer was significant in the upper (Suppl Fig. 2a, b) and lower small intestinal tissues of vaccinated male mice (Suppl Fig. 2c, d). Furthermore, α-GalCer significantly increased bacteria- and CTB-specific serum IgA (Suppl Fig. 1c, d) as well as CTB-specific serum IgG1 titres (Suppl Fig. 1f) compared to Dukoral^®^ alone. A minor increase in bacteria-specific serum IgG1 responses was also observed (Suppl Fig. 1e).

**Fig. 2:**
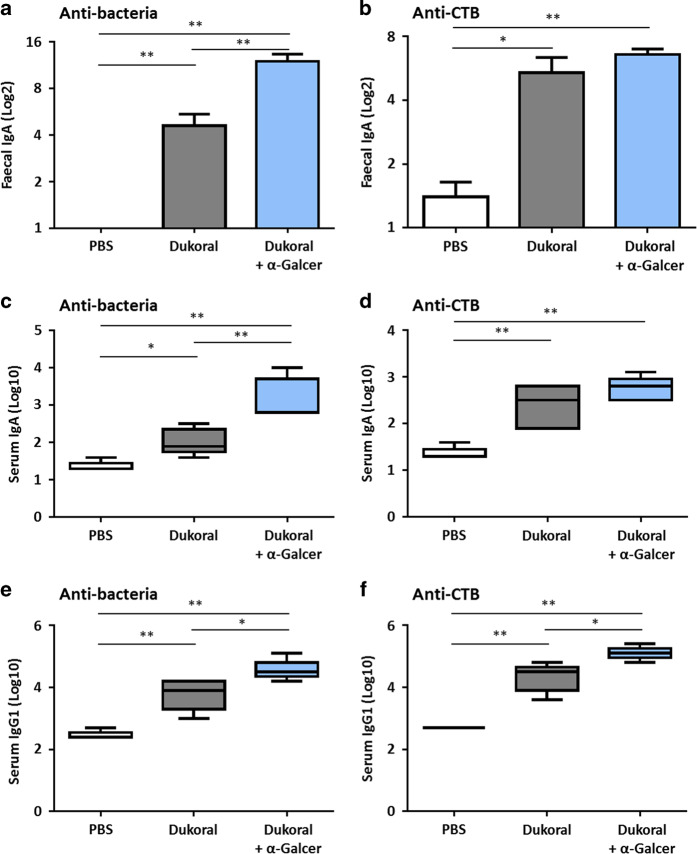
α-GalCer enhances antigen-specific faecal IgA, serum IgA and IgG1 responses after oral vaccination of female BALB/c mice. Mice were immunized orally with Dukoral^®^ either with or without α-GalCer as an adjuvant. Faecal pellets and sera were collected on days 41 and 42, respectively. Bacteria-specific and CTB–specific IgA responses in faecal pellets (**a**, **b**) and serum (**c**, **d**) as well as bacteria-specific and CTB-specific IgG1 responses in serum (**e**, **f**) were measured and antibody titres were determined by end-point ELISA. Graphs present mean titres (+SEM) for five mice per experimental group. *p < 0.05, **p < 0.01

In female BALB/c mice, the administration of α-GalCer with Dukoral^®^ significantly enhanced bacteria-specific intestinal IgA responses (Fig. 2a) in FPS as well as bacteria-specific IgA (Fig. 2c) and IgG1 responses in serum (Fig. 2e). Vaccination with Dukoral^®^ alone induced high CTB-specific intestinal IgA titres in FPS (Fig. 2b) and serum IgA (Fig. 2d), which was slightly but not significantly enhanced in mice vaccinated with Dukoral^®^ and α-GalCer. However, the vaccine containing α-GalCer significantly increased CTB-specific IgG1 responses in serum compared to Dukoral^®^ alone (Fig. 2f).

### α-GalCer enhanced intestinal Th1 responses after oral vaccination in C57BL/6 mice

**Fig. 3:**
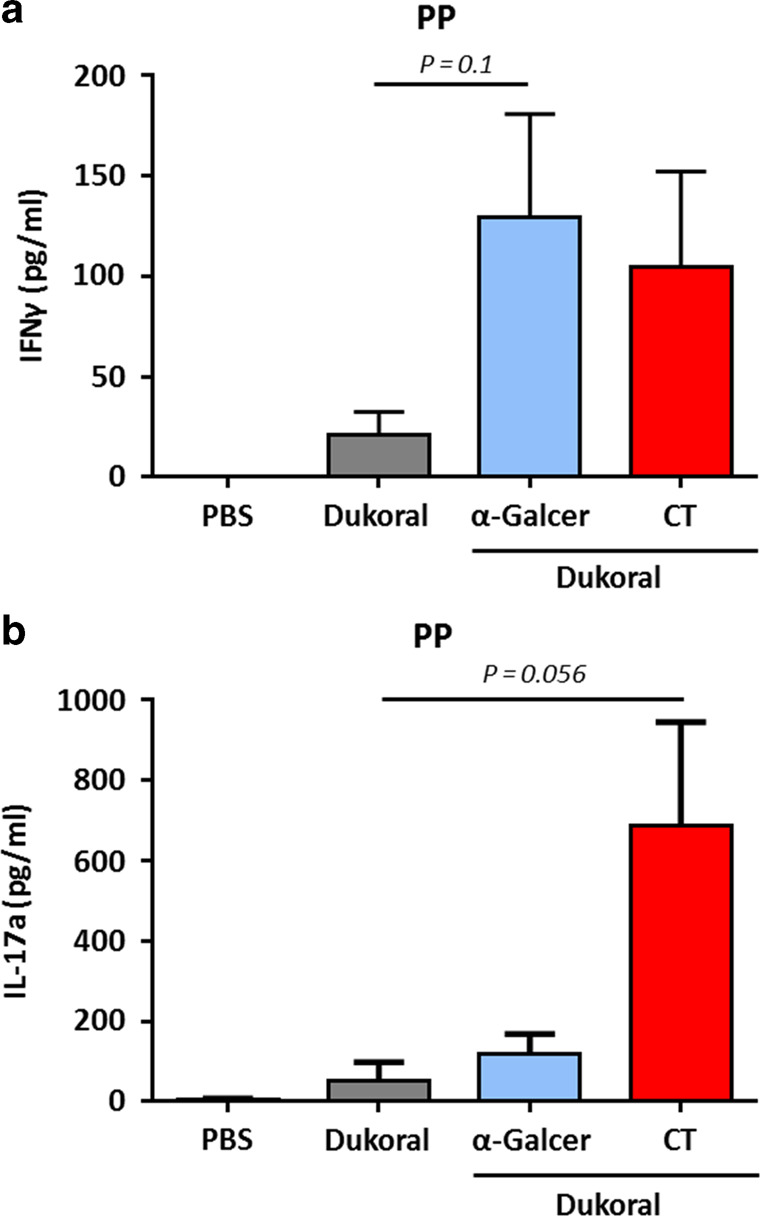
α-GalCer promotes Peyer’s patch Th1 but not Th17 responses after oral vaccination in female C57BL/6 mice. Mice were immunized orally with Dukoral^®^ either with or without α-GalCer or CT as an adjuvant. Peyer’s patch (PP) cells were processed on day 42 and stimulated with 4 × 10^6^ Dukoral^®^ bacteria. Seventy-two hours post-stimulation, IFNy (**a**) and IL-17A (**b**) secretion was measured in supernatants by ELISA. Graphs present IFNy and IL-17A concentrations in supernatants (+SEM) for five mice per experimental group

Even though *V. cholerae* is an extracellular non-invasive pathogen against which protection is largely antibody-mediated, it was shown that T cell-mediated responses were induced after cholera infection.^12,13^ Th1 and Th17 responses were observed after ex vivo stimulation of whole blood from naturally infected adults with a *V. cholerae* membrane preparation. In addition, IL-17 responses were observed in intestinal biopsies of patients after natural *V. cholerae* O1 infection. However, comparable Th1 and Th17 responses were not observed after vaccination with Dukoral^®^, which could contribute to the short duration of protection after administration of OCVs.^14^ Furthermore, it was clearly demonstrated that CD4^+^ T cell responses were crucial to induce protective antigen-specific intestinal IgA responses following oral immunization with the powerful mucosal immunogen CT in mice^15^ and interestingly, IFNγ-R signaling promoted antigen specific IgA responses.^16^ Based on this knowledge, the benefits of using α-GalCer as an adjuvant to promote Dukoral^®^-specific T cell-mediated responses was also evaluated. Strikingly, Peyer's patch (PP) cells from mice vaccinated with Dukoral^®^ adjuvanted with α-GalCer showed higher production of IFNγ after restimulation with Dukoral^®^ bacteria compared to similarly stimulated PP cells from mice vaccinated with Dukoral^®^ alone (Fig. 3a). Immunization with Dukoral^®^ and α-GalCer did not significantly enhance splenic or MLN Th1 responses compared to Dukoral^®^ alone (Suppl Fig. 3a, b).

Contrary to CT, which enhanced PP Th1 and Th17 responses, α-GalCer did not enhance PP Th17 responses (Fig. 3b). CT also enhanced splenic and MLN Th17 responses although the difference over Dukoral^®^ alone was not significant (Suppl Fig. 5a, b). Restimulation of PP cells with Concanavalin A (ConA) revealed that vaccination with Dukoral^®^ and α-GalCer primed PP cells for enhanced IFNγ production while PP cells from mice that received Dukoral^®^ and CT were primed for enhanced IFNγ and IL-17 production (Suppl Fig. 4a and Suppl Fig. 6a). These effects were not seen in the case of spleen and MLN cells suggesting a local nonspecific response to the adjuvant (Suppl Fig. 4b, c and Suppl Fig. 6b, c). Th1 responses were also analyzed in male C57BL/6 after immunization with Dukoral^®^ alone or adjuvanted with α-GalCer. Similarly to female C57BL/6 mice, PP cells from male C57BL/6 mice vaccinated with Dukoral^®^ and α-GalCer showed higher production of IFNγ after restimulation with Dukoral^®^ bacteria compared to cells from males vaccinated with the Dukoral® alone (Suppl Fig. 7a). Similar results were observed after restimulation with ConA (Suppl Fig. 8a). As observed in female C57BL/6 mice, no increase of IFNγ production was measured in splenocytes and MLN cells stimulated with Dukoral^®^ bacteria (Suppl. Fig. 7b, c) or Con A (Suppl. Fig. 8b, c) from males immunized with the vaccine adjuvanted with α-GalCer compared to Dukoral^®^ alone. No intestinal and systemic Th1 responses were detected in BALB/c mice (data not shown).

### α-Galcer increases intestinal and systemic antibody responses to a novel double-LPS expressing WCK *V. cholerae* Hikojima strain

**Fig. 4:**
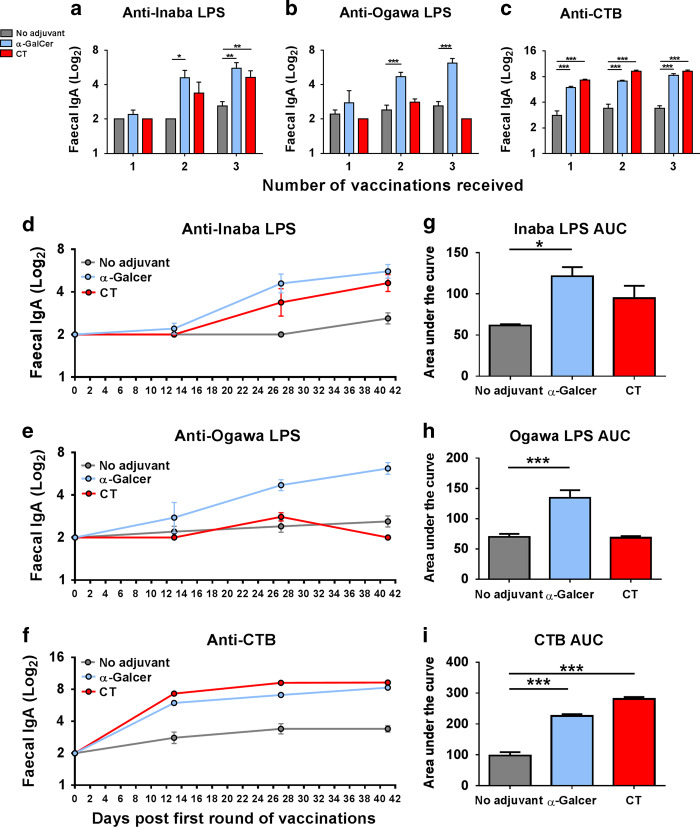
α-GalCer enhances LPS and CTB-specific faecal IgA responses compared to the oral vaccine with MS1342 *V. cholerae/*CTB alone. Mice were immunized orally with MS1342 *V. cholerae* and CTB either with or without α-Galcer or CT as an adjuvant. Faecal pellets were collected and **a**, **d** Inaba LPS-specific, **b**, **e** Ogawa LPS–specific and **c**, **f** CTB-specific IgA antibody titres were determined by end-point ELISA. Panels (**d–f**) present mean titres (+SEM) for five mice per experimental group at three time-points. Graphs (**g**–**i**) present the mean areas under the curve (AUC) (+SEM) for each experimental group. No adjuvant versus α-GalCer or CT: *p < 0.05, **p < 0.01, ***p < 0.001

**Fig. 5:**
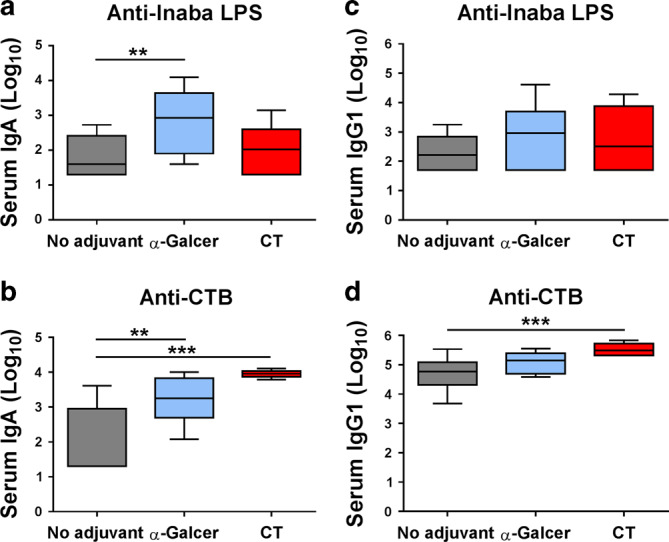
α-GalCer enhances serum LPS and CTB-specific IgA titres compared to the oral vaccine with MS1342 *V. cholerae/*CTB alone. Mice were immunized orally with MS1342 *V. cholerae* and CTB either with or without α-GalCer or CT as an adjuvant. Serum was collected on day 41. Inaba LPS –specific (**a**, **c**) and CTB-specific (**b**, **d**) IgA as well as IgG1 antibody titres were determined by end-point ELISA. Graphs present mean titres (+SEM) for five mice per experimental group. No adjuvant versus α-GalCer or CT: **p < 0.01, ***p < 0.001

Dukoral^®^ is composed of four strains of *V. cholerae* expressing either Inaba or Ogawa LPS, inactivated by different procedures. Issues relating to the complexity and cost of production, storage and distribution hampers the widespread public health application of Dukoral^®^^17^. However, the novel double-LPS expressing WCK *V. cholerae* Hikojima strain (MS1342)^5,6^ could be an effective way to simplify OCV production and enhance cost-effectiveness. We evaluated the ability of α-GalCer to enhance intestinal antigen-specific IgA responses to MS1342. This strain was administered orally with CTB alone or adjuvanted with α-GalCer or CT. Oral co-administration of α-GalCer significantly increased IgA responses against Inaba and Ogawa LPS compared to non-adjuvanted MS1342/CTB in FPS after each round of immunization (Fig. 4a, b). Interestingly, intestinal Ogawa LPS responses were also higher with MS1342/CTB and α-GalCer compared to the vaccine formulation containing CT (Fig. 4b). In addition, co-administration of α-GalCer significantly enhanced IgA responses against CTB, as measured in FPS, compared to non-adjuvanted MS1342/CTB (Fig. 4c) comparable to the strong responses obtained with MS1342/CTB and CT after each round of immunization. When the area under the curve (AUC) was analyzed, a measure of the rate of increase in antibody titres over time,^8^ α-GalCer significantly enhanced anti-Inaba (Fig. 4d, g) and Ogawa LPS (Fig. 4e, h) IgA titres above that of the CT-adjuvanted MS1342/CTB, while a trend where CT preferentially enhanced anti-CTB IgA AUC was observed (Fig. 4f, i). Importantly, this pattern was replicated for anti-Inaba and anti-CTB IgA titres in the serum. It is noteworthy that, in contrast to the intestinal responses, oral vaccination with MS1342 and CTB more effectively promoted serum antibody responses. α-GalCer was an effective adjuvant for promoting serum IgA anti-Inaba LPS responses compared to non-adjuvanted MS1342/CTB (Fig. 5a) and promoted comparable anti-CTB IgA responses to those induced by MS1342/CTB and CT (Fig. 5b). However, when serum IgG1 was analyzed, only CT significantly enhanced CTB-specific titres (Fig. 5d), while no differences were found between the ability of CT or α-GalCer to enhance anti-Inaba LPS IgG1 titres (Fig. 5c).

### SmPill^®^ minispheres can be accurately loaded with WCK bacteria and subunit antigens

**Fig. 6:**
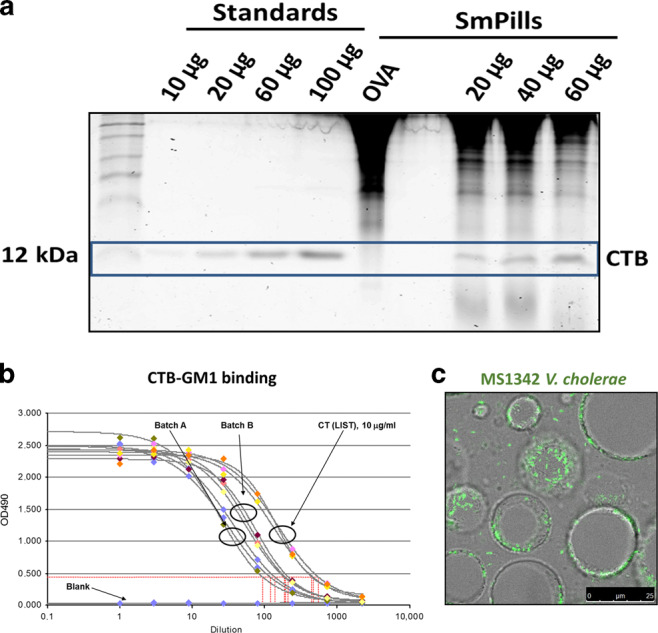
The SmPill^®^ manufacturing technique can ensure accurate and intact loading of vaccine formulation components. SmPill^®^ minispheres were dissolved in PBS at 50 °C for 1 h. **a** SmPill^®^ supernatants and CTB of known concentration were run on an SDS-polyacrylamide gel and stained with Coomassie blue. Bands corresponding to CTB can be found at 12 kDa and show that SmPill^®^ can be accurately loaded with rCTB. **b** Following dissolution of SmPill^®^ minispheres from two batches, GM1-specific ELISA was run in triplicate to confirm the binding capacity of rCTB inside the minispheres. CT in solution was shown as a technical positive control. The red dotted line represents the technical cut-off. **c** Confocal microscopy of SmPill^®^ minisphere sections containing GFP-tagged *V. cholerae* reveal loading of these into the dispersed phase droplets in the superstructure of minispheres

We recently demonstrated that the incorporation of WCK *E. coli* over-expressing ETEC colonization factors co-formulated with α-GalCer in SmPill^®^ enhanced the ability of this vaccine to induce intestinal IgA responses.^8^ Several oral drug delivery technologies have been evaluated as vehicles to enable oral vaccination. However, most have been abandoned as sensitive vaccine components, especially subunit antigens, are degraded during the manufacturing process.^9^ In order to ensure that CTB, the key antigen for anti-toxin immunity, could be incorporated intact into SmPill^®^ minispheres, we developed an assay to effectively test the incorporation of intact rCTB into SmPill^®^ minispheres. When beads loaded with 20 µg and 60 µg CTB respectively were dissolved in PBS and the supernatants run on an SDS-PAGE gel, which was then stained with Coomassie dye and imaged, CTB (ca 12 kDa) bands appeared at similar intensities to corresponding bands from 20 µg and 60 µg CTB standards (Fig. 6a) indicating efficient recovery of the encapsulated CTB. To insure the intactness of CTB within the SmPill^®^ minispheres, its binding capacity was tested by GM1-binding ELISA assay in two batches of minispheres. The assay showed that CTB after encapsulation retained its ability to bind the GM1 receptor. CT was used here as a positive control (Fig. 6b). Finally, in order to localize the MS1342 *V. cholerae* strain in SmPill^®^ minipheres, beads containing MS1342 bacteria expressing green fluorescent protein (GFP) were formulated, sectioned and imaged using confocal microscopy. We found the majority of GFP-expressing MS1342 bacteria to be localized in the oil phase droplets inside the hydrophilic SmPill^®^ matrix (Fig. 6c), which is consistent with our previous work.^8^

### A novel oral cholera vaccine formulation encapsulated in SmPill^®^ minispheres induces stronger intestinal antigen-specific IgA responses than Dukoral^®^

**Fig. 7:**
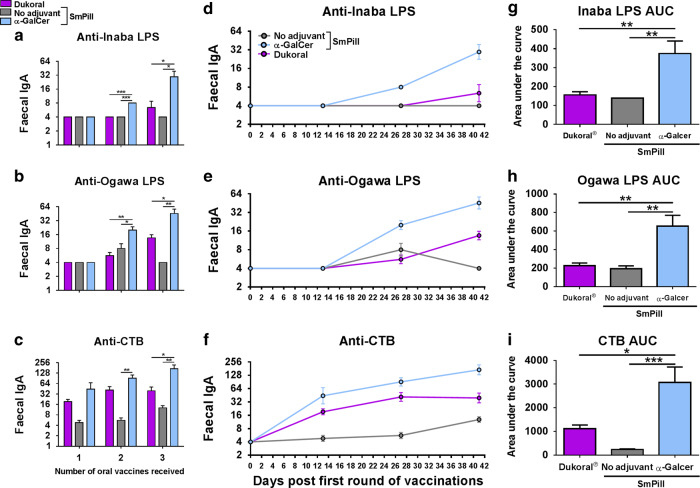
SmPill^®^ containing whole-cell killed MS1342 *V. cholerae* together with CTB and α-GalCer elicit stronger faecal antigen-specific IgA titres than Dukoral^®^. Mice were immunized orally with SmPill^®^ containing CTB and WCK MS1342 *V. cholerae* with or without α-GalCer as an adjuvant, or with Dukoral^®^ in solution. Faecal pellets were collected on days 13, 27 and 41 and **a** Inaba-specific, **b** Ogawa LPS–specific and **c** CTB-specific IgA antibody titres were determined by end-point ELISA. Panels (**d**–**f**) present mean titres (+SEM) for five mice per experimental group at three time-points. Graphs (**g**–**i**) present the mean areas under the curve (AUC) (+SEM) for each experimental group. Dukoral^®^ versus SmPill®/no adjuvant versus α-GalCer: *p < 0.05, **p < 0.01, ***p < 0.001

The ability to incoporate antigens such as the WCK *V. cholerae* Hikojima strain MS1342 and CTB within SmPill^®^ minispheres was confirmed. The following step was to compare side-by-side immune responses in mice induced after oral vaccination with SmPill^®^ loaded with MS1342/CTB and α-GalCer in comparison to responses generated with Dukoral^®^ in solution. Strikingly, the α-GalCer adjuvanted SmPill^®^ vaccine formulation induced significantly higher anti-Inaba LPS (Fig. 7a), anti-Ogawa LPS (Fig. 7b) and anti-CTB (Fig. 7c) IgA titres in FPS than vaccination with either Dukoral^®^ alone or non-adjuvanted antigen-containing SmPill^®^ minispheres. Likewise, the α-GalCer adjuvanted SmPill^®^ vaccine formulation gave significantly higher AUC values compared to Dukoral^®^ or non-adjuvanted antigen-containing SmPill^®^ minispheres for both Inaba LPS (Fig. 7d, g) and Ogawa LPS (Fig. 7e, h) as well as CTB (Fig. 7f, i) IgA responses, thus confirming that α-GalCer adjuvanted antigen-containing SmPill^®^ induces not only significantly higher antigen-specific titres but also that such titres are achieved at a faster rate compared to Dukoral^®^ or non-adjuvanted antigen-containing SmPill^®^ minispheres (Fig. 7).

Interestingly and similar to what we observed for ETEC,^8^ the incorporation of WCK *V. cholerae* Hikojima strain MS1342 and CTB within SmPill^®^ minispheres in the absence of α-GalCer did not enhance antigen-specific serum IgA (Suppl Fig. 9a, b) or serum IgG1 (Suppl Fig. 9c, d). However, the addition of α-GalCer within the antigen-containing SmPill^®^ minispheres resulted in antigen-specific serum IgA (Suppl Fig. 9a, b) and serum IgG1 (Suppl Fig. 9c, d) responses that were comparable to those induced by Dukoral^®^ alone.

## Discussion

In October 2017, the Global Task Force on Cholera Control, a network composed of UN and international agencies, academic institutions, and NGOs that supports countries affected by the disease, launched an ambitious plan to reduce cholera deaths by 90% by 2030. The current strategies to prevent disease transmission and control cholera outbreaks include programs to improve access to safe drinking water, sanitation and hygiene and the use of OCVs.^18^ Currently, three OCVs have been licensed and prequalified by WHO: Dukoral^®^, Shanchol™ and Euvichol™. Even though the efficacy of OCVs has been clearly demonstrated in clinical and field trials, their production is complex and expensive. Furthermore, it is a priority to increase the duration of protective immunity, especially in young children.^19^ Consequently, there is an urgent need to address challenges related to long-term efficacy, administration and storage of OCVs in an integrated manner.

This study demonstrates that the addition of the oral adjuvant α-GalCer could enhance mucosal immunogenicity of the internationally most widely licensed OCV, Dukoral^®^. Indeed α-GalCer significantly increased intestinal anti-LPS and anti-CTB IgA responses known to be important for immune protection against *V. cholerae* infections. In addition, increased serum anti-LPS and anti-CTB IgA and IgG1 responses were observed. Interestingly, α-GalCer also promoted intestinal Th1 responses to a greater extent than Dukoral^®^ alone. T cell responses have been demonstrated to be different after a natural *V. cholerae* infection and after vaccination,^20^ and lower T cell and memory B cell responses induced by current OCVs might limit duration of protection after vaccination compared with immunity after natural infection and disease.^14,21^ The addition of α-GalCer might be beneficial to induce long-term protection againt *V. cholerae*, particularly at the site of infection, after administration of OCVs. The efficacy of oral α-GalCer administration was also addressed in male C57BL/6 mice. We observed that α-GalCer enhanced intestinal and systemic anti-LPS and CTB-specific responses but to a lower extent than that seen in females. However, intestinal Th1 responses were similar in both sexes. Sex has been documented to significantly impact on vaccination outcomes^22^ but this has received relatively little attention in the context of oral vaccination. There is evidence that the number of iNKT cells and their cytokine production is higher in women^23^ and female mice.^24^ While a detailed analysis is outside the scope of this study, future work will investigate these differences in antibody responses and address whether adjustments in oral antigen and adjuvant dose can overcome sex differences in responses. Furthermore, it is known that the genetic background influences the antibody and cellular immune responses to vaccines.^25^ Here oral α-GalCer administration was particularly effective in enhancing intestinal and systemic anti-LPS antibody responses in BALB/c mice but did not promote detectable antigen-specific intestinal and systemic Th1 responses. Consequently, the enhancement of antigen-specific intestinal IgA responses by α-GalCer appears to be independent of antigen-specific Th1 responses.

In order to simplify the production of OCVs, the immunogenicity of a novel formalin killed Hikojima O1 *V. cholerae* MS1342 strain constitutively co-expressing both the Ogawa and Inaba O1 LPS antigens^5^ administered with rCTB was evaluated. Cost-effective OCV production using a single-strain formulation rather than the several bacterial strains and formulations used in currently licensed WCK OCVs would be a major advance. Oral vaccination with MS1342 *V. cholerae* elicited intestinal and systemic anti-Inaba and anti-Ogawa LPS antibody responses.^5^ The addition of α-GalCer further increased anti-LPS faecal-IgA titres compared to both the antigen alone and even the antigen given together with the “gold standard” mucosal adjuvant CT. These results suggest that α-GalCer is a particularly effective inducer of immunity to antigens delivered on WCK bacteria, which is consistent with our previous findings.^8^ Furthermore, α-GalCer significantly enhanced anti-CTB faecal IgA titres compared to the non-adjuvanted formulation. The addition of α-GalCer to the WCK bacteria and CTB vaccine also greatly enhanced the rate at which IgA titres were established over time, even compared to the antigen with CT.

In addition to its impressive oral adjuvanticity in mice, α-GalCer has been tested in clinical trials in the context of therapeutic strategies for cancer and hepatitis in which its safety has been assessed. After intravenous injection of α-GalCer in advanced cancer patients^26^ or patients with chronic hepatitis B/C,^27,28^ only minimal and transient side effects were observed. Clinical trials were also performed with α-GalCer-pulsed DCs administered to patients by intravenous,^29,30^ nasal, submucosal^31,32^ or intradermal^33^ injections with no major side effects reported. In humans, α-GalCer has not yet been tested as a vaccine adjuvant by the oral route but based on our current results this should be a priority.

Talavera and colleagues demonstrated that coating of WCK *V. cholerae* incorporated in polymer micro-particles (MPs), with Eudragit^®^ L-30 D-55 enhanced the ability of MPs to resist degradation in acidic medium but also provided a controlled release function.^34,35^ In the current study, we evaluated the potential of the oral delivery technology, SmPill^®^ to enhance OCV effectiveness. This system has completed a Phase II clinical trial, delivering cyclosporine to the colon for the local treatment of ulcerative colitis (NCT01033305).^36^ In the context of oral vaccine delivery, the incorporation of oral vaccine formulation in SmPill^®^ minispheres was demonstrated to improve the efficacy of an oral ETEC candidate vaccine.^8^ When MS1342 *V.cholerae* and CTB were incorporated into SmPill^®^, we were able to recover intact CTB with preserved GM1-binding capacity indicating maintenance of antigenic stability. The ability to produce capsules containing pre-dosed minispheres would greatly enhance the effectiveness of mass vaccination in response to disease outbreaks and simplify mass vaccination campaigns in endemic regions. Consistent with our previous findings,^8^ we were able to show loading of WCK bacteria into the dispersed phase of the SmPill^®^ indicating that the technology platform can be loaded with diverse WCK bacterial strains. Finally, we compared SmPill^®^ containing WCK MS1342 *V. cholerae* co-formulated with rCTB and α-GalCer to the WHO licensed OCV Dukoral^®^. Our adjuvanted SmPill^®^ OCV formulation out-performed Dukoral^®^ in its ability to enhance intestinal IgA responses against both Inaba and Ogawa LPS in addition to CTB-specific IgA responses. In humans the intestinally induced IgA anti-LPS and anti-CTB antibody responses as measured in intestinal lavage fluid have correlated closely to the protective efficacy of cholera vaccines.^37–39^ Even though vaccine-induced protection against intestinal *V. cholerae* infection cannot be measured in immunocompetent adult mice, our findings would strongly suggest that such adjuvantation if achievable with α-GalCer and SmPill^*®*^ in humans could benefit the protective immunogenicity of OCVs. Furthermore, SmPill^®^ also induced significantly faster establishment of antibody responses compared to Dukoral^®^. These results support the use of integrated strategies, comprising novel WCK antigens, powerful mucosal adjuvants and delivery systems, such as MS1342 *V. cholerae*, α-GalCer and SmPill^®^, to enhance both the immunogenicity of OCV formulations and the speed at which antibody responses are established over time, making these strategies amenable to both short term protection from Cholera outbreaks and the long term goal of cholera elimination.

## Methods

### Animals

Female and male C57BL/6 as well as BALB/c mice were obtained from Charles River Laboratories, Inc., and were used at 12–16 weeks of age. Animals were maintained according to the regulations of the EU and the Irish Department of Health and all procedures performed were conducted under animal licence number B100/3321 and were approved by the Trinity College Dublin Animal Research Ethics Committee (Ethical Approval Number 091210).

### Immunization strategy

Groups of mice (*n* = 5) were immunized orally in three rounds on days 0 and 1; 14 and 15, and 28 and 29. One hour prior to immunization, food was withdrawn. Mice were gavaged with 200 µl of solution containing 100 µl of 0.3 M sodium bicarbonate buffer (pH 9) and 100 µl Dukoral^®^ with or without α-GalCer (10 μg) (Avanti Lipids) or CT (10 μg) (LIST Biologicals), or with PBS alone. Alternatively, mice were gavaged with 200 µl of 0.3 M sodium bicarbonate buffer. After 20 minutes, mice were gavaged with 200 µl of sterile PBS containing 3 × 10^8^ WCK MS1342 bacteria and 30 µg CTB per mouse with or without α-GalCer (10 μg) (Avanti Lipids) or CT (10 μg) (LIST Biologicals) or with PBS alone. Alternatively, mice were anesthetized with Isoflurane and vaccinated with three SmPill^®^ minispheres which were delivered by loading the minispheres into the silicon tip of a 17 G flexible feeding tube (Agntho’s, Sweden) and 50 µl of 1X PBS adjusted to pH 5.

### Collection of faecal, serum and intestinal samples

Faecal pellets, blood and intestinal tissues were collected and processed as described previously.^8,40^

### ELISA for measurement of antigen-specific antibody responses

For CTB-specific responses, medium-binding 96-well plates (Greiner BioOne) were coated with 50 μl per well of 0.3 nmol/ml Bovine GM1 (Sigma Aldrich) in 1X PBS and incubated overnight at 4 °C. Plates were washed once in 1X PBS and blocked with 200 μl 0.1% BSA for 30 mins at 37 °C. The blocking solution was washed from the plates once with 1X PBS and 50 μl per well of 0.5 μg/ml CTB (Sigma Aldrich) was added and incubated for 60 min at room temperature (RT). The plates were then washed three times in wash buffer (PBS-T: PBS 1 × /0.05% Tween (Sigma Aldrich)). For LPS-specific titres, plates were coated with 50 μl per well of 5 μg/ml Inaba or Ogawa LPS diluted in 1X PBS and incubated overnight at 4 °C. For Dukoral^®^ bacteria-specific plates, the vaccine formulation was centrifuged to separate the bacteria and rCTB and 4 × 10^6^ bacteria diluted in 50 µl of 1X PBS were added to each well, then incubated overnight at 4 °C. Plates were washed three times in 1X PBS and blocked with 200 μl 0.1% BSA for 60 minutes at 37 °C. 50 µl of sample (FPS or serum) was added and serially diluted across the plate in 0.1% BSA/PBS-T and incubated overnight at 4 °C. After washing three times in wash buffer, 50 μl per well of HRP-conjugated anti-mouse IgA (1:1000) (Southern Biotech) or biotin-conjugated IgG1 (1:4000) (BD Pharmingen) was added to each well and incubated for 2 h at RT. Plates were washed four times in wash buffer and for the IgG1 ELISA, 50 µl per well of Streptavidin-HRP (1:2000) (BD Pharmingen) was added to each well and plates were incubated for 30 min at RT. Plates were washed four times in wash buffer and one final time in 1X PBS. One milligram per milliliter o-Phenylenediamine dihydrochloride substrate (Sigma Aldrich) was prepared in 0.1 M phosphate citrate buffer (pH 5) containing 4 μl H_2_O_2_ per 10 ml substrate and 50 μl added per well. The plates were left to develop at RT in the dark, the reaction was then stopped by the addition of 25 μl/well of 1 M H_2_SO_4_ and the absorbance at 492 nm read using a Microplate Reader (Thermo Scientific) running Scan-IT software (Thermo Scientific) to acquire data. Antibody concentrations were expressed as endpoint titres calculated by regression of a curve of OD values versus reciprocal sample levels to a cut-off point of 2 or 3 standard deviations above control samples.

### Measurement of cellular responses

Mice were sacrificed by cervical dislocation before removal of spleens, mesenteric lymph nodes (MLNs) and Peyer’s patches (PPs). An enzymatic treatment had to be first performed in order to prepare PP cell suspensions. PPs were cut into small pieces and incubated in 1 ml RPMI 1640 medium/1% HEPES (Gibco, Life Technologies) containing 42 µg/ml Liberase TM Research Grade (Roche 5401119001), 0.5 mg/ml DNase I from bovine pancreas (Roche, 11284932001) and 2 mM CaCl_2_ (Sigma Aldrich) for 30 min at 37 °C/5%CO_2_. In order to stop the enzymatic reaction, 2 ml of cold complete RPMI 1640 medium (cRPMI) (Gibco, Life Technologies) were added. cRPMI is RPMI 1640 medium supplemented with 1% Penicillin/Streptomycin 100 × (Gibco Life Technologies), 1% L-glutamine 200 mM (Gibco Life Technologies) and 10% heat-inactivated and filtered Fetal Bovine Serum (Biosera). Single cell suspensions of PP cells, MLN cells and splenocytes were prepared by disrupting tissue through 40 µm nylon cell strainers (BD Falcon) with cRPMI. After centrifugation of MLN and PP cells, they were resuspended in 1 ml of enriched RPMI. Enriched RPMI is RPMI 1640 medium supplemented with 10% heat-inactivated and filtered Fetal Bovine Serum (Biosera), 1% sodium pyruvate 100 mM 100 × (Gibco Life Technologies), 1% Non-essential amino acids 100 × (Gibco Life Technologies), 1% L-glutamine 200 mM (Gibco Life Technologies), 0.1% Penicillin/Streptomycin 100 × (Gibco Life Technologies), 0.1% Mercaptoethanol 55 mM (Gibco Life Technologies), 0.4% MEM vitamins (Gibco Life Technologies). For red blood cell lysis in splenocyte suspensions, cell pellets were resuspended in 1 ml 0.88% ammonium chloride solution (Sigma Aldrich) for 2 min and washed in cRPMI medium before resuspending the pellets in 5 ml enriched RPMI. Splenocytes were plated at 4 × 10^5^ cells/well and MLN and PP cells at 2 × 10^5^ cells/well in 100 µl of enriched RPMI.

Cells were stimulated for 72 h at 37 °C/5%CO_2_ with the following stimuli in 100 µl of enriched RPMI 1640 medium: Concanavalin A (Con A) (4 µg/ml, Sigma Aldrich) or Dukoral^®^ bacteria without rCTB (4 × 10^5^/well bacteria for splenocytes, 1.8 × 10^6^/well or 4 × 10^6^/well bacteria for MLN cells and PP cells). IFNγ and IL-17A release in supernatants was measured post-stimulation using Mouse IFNγ and IL-17A DuoSet ELISA kits (R&D systems).

### Manufacture of SmPill^®^

SmPill^®^ minispheres were manufactured as previously detailed.^8,41^ Three SmPill^®^ contained 3 × 10^8^ formalin-killed *V. cholerae* Hikojima MS1242 bacteria,^5^ 30 µg rCTB and 10 µg of α-GalCer when required. SmPill^®^ were coated with Eudragit L-30 D-55 (Evonik, Industries AG, Germany).

### SDS PAGE analysis of CTB from SmPill^®^

Three SmPill^®^ minispheres were dissolved in 1 ml of PBS 1X at 50 °C for 1 h. Supernatants from dissolved SmPill^®^ and CTB standards were mixed with denaturing sample buffer. Samples were boiled for 3 mins and 20 μl of sample was loaded into each well in a 15% gel in addition to a multiprotein molecular weight reference (Dual Colour 5–75 kDa, Biorad). The gel was run at 120 V for 90 mins in running buffer 1 × (10 × : 250 mM Tris, 1.92 M Glycine, 1% Sodium Dodecyl Sulfate) and stained with Coomassie Blue overnight at RT. The next day, gels were washed with destaining solution and the gel was scanned on an Osiris gel scanner.

### ELISA for analysis of CTB integrity in SmPill^®^

The coating was performed as for the CTB-specific ELISA outlined above. SmPill^®^ minispheres were dissolved as previously described, the solutions were serially diluted and 50 μl per well of solutions were added to the ELISA plates, then incubated for 2 h at RT. The plates were then washed three times in PBS-T and 50 μl of anti-CTB antibodies per well was added for 60 min at RT. After three times washing in wash buffer, 50 µl of goat anti-mouse IgG HRP (Jackson ImmunoResearch, 115–035–062) was added to each well for 30 min at RT. Finally, plates were washed four times in wash buffer and one final time in 1X PBS, then developed as previously described.

### Microscopic analysis of SmPill^®^

SmPill^®^ containing GFP-tagged *V. cholerae* were embedded in TissueTek OCT compound (Sakura Finetek) and snap frozen in liquid nitrogen. Sections (7 µm) were observed using the Olympus FV1000 scanning confocal microscope (SBI microscopy facility, TBSI).

### Statistics

The data represented in each graph are arithmetic means. Analysis of variance (ANOVA) was used to determine significant differences between treatments, and the degree of any significance was calculated by Tukey’s multiple comparison test. When required, a Mann–Whitney test was used to determine the degree of significance between two specific treatments. Prism5 (GraphPad) was used for all statistical analysis. *P* values < 0.05 were regarded as significant.

## Supplementary information

Supplementary Material
