# Supplementary material for: Alpha-galactosylceramide enhances mucosal immunity to oral whole-cell cholera vaccines
Source: Mucosal Immunol. 2019 Apr 5;12(4):1055–64. doi: 10.1038/s41385-019-0159-z (PMC7746523; doi:10.1038/s41385-019-0159-z)

**Title:**

**Alpha-Galactosylceramide enhances mucosal immunity to oral whole-cell cholera vaccines**

**Running Title:**

**Novel adjuvanted oral whole-cell cholera vaccine**

**Authors:**

Christopher JH Davitt^1*^, Stephanie Longet^1*^, Aqel Albutti^1,2^, Vincenzo Aversa^3^, Stefan Nordqvist^4^, Becky Hackett^1^, Craig P McEntee^1^, Monica Rosa^3^, Ivan S Coulter^3^, Michael Lebens^4^, Joshua Tobias^4^, Jan Holmgren^4^ and Ed C Lavelle^1,5^.

^1^Adjuvant Research Group, School of Biochemistry and Immunology, Trinity Biomedical Sciences Institute, Trinity College Dublin, Dublin 2, D02 R590, Ireland.

^2^College of Applied Medical Sciences, Qassim University, Buraydah 52571, Saudi Arabia.

^3^Sublimity Therapeutics (Holdco) Ltd, DCU Alpha Innovation Campus, Old Finglas Road, Dublin, D11 KXN4 Ireland.

^4^University of Gothenburg Vaccine Research Institute (GUVAX), Dept. of Microbiology and Immunology, University of Gothenburg, Box 435, 405 30 Gothenburg, Sweden.

^5^Centre for Research on Adaptive Nanostructures and Nanodevices (CRANN) & Advanced Materials Bio-Engineering Research Centre (AMBER), Trinity College Dublin, Dublin 2, D02 PN40, Ireland.

******* these authors contributed equally to this work

**Corresponding author:**

Prof Ed C. Lavelle

Adjuvant Research Group

School of Biochemistry and Immunology

Trinity Biomedical Sciences Institute

Trinity College Dublin,

D02 R590, Ireland.

Email: lavellee@tcd.ie

Phone: 353 1 8962488

**Keywords**

oral vaccine; adjuvant; enteric delivery system; cholera; mucosal immunity


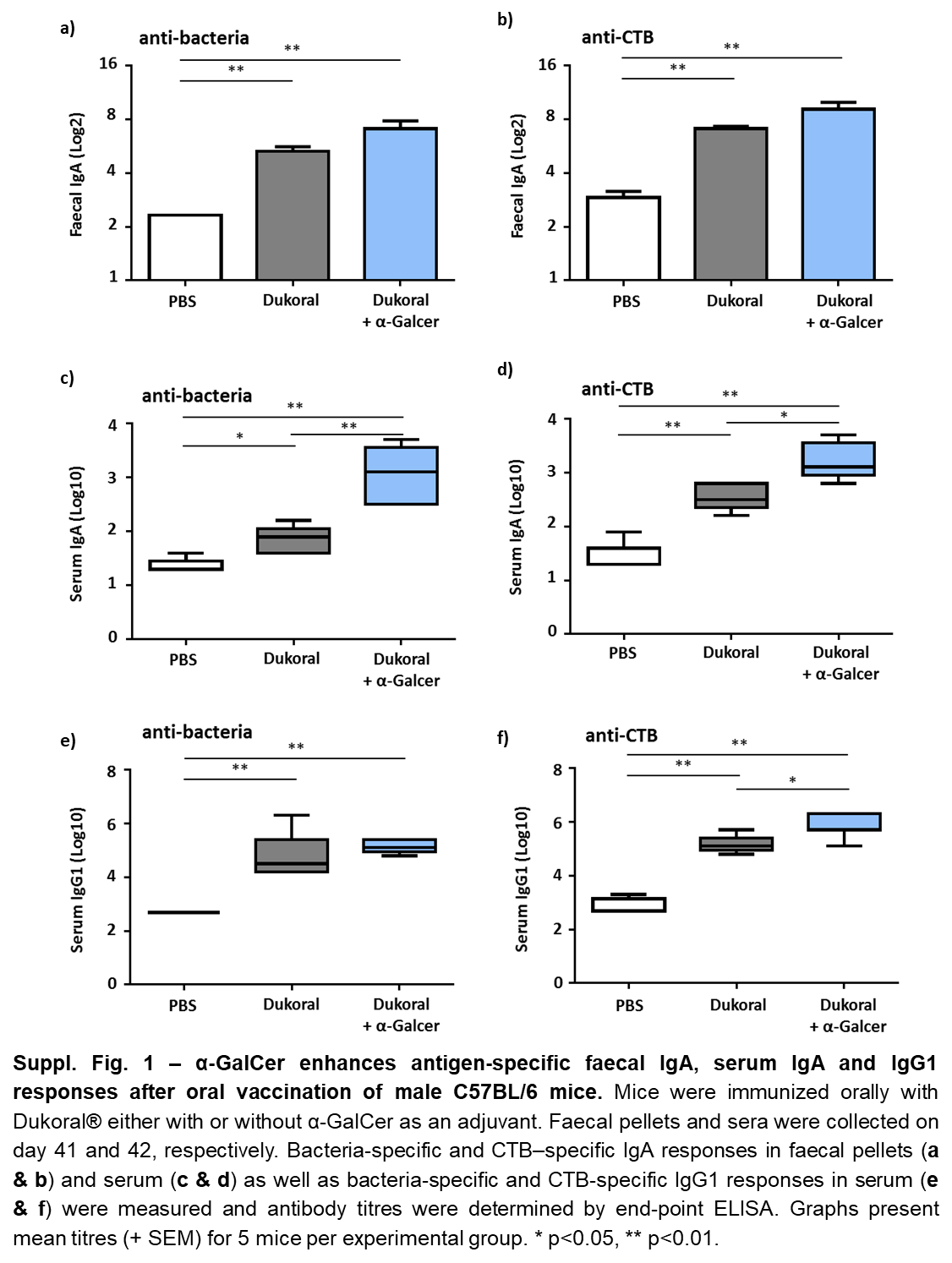


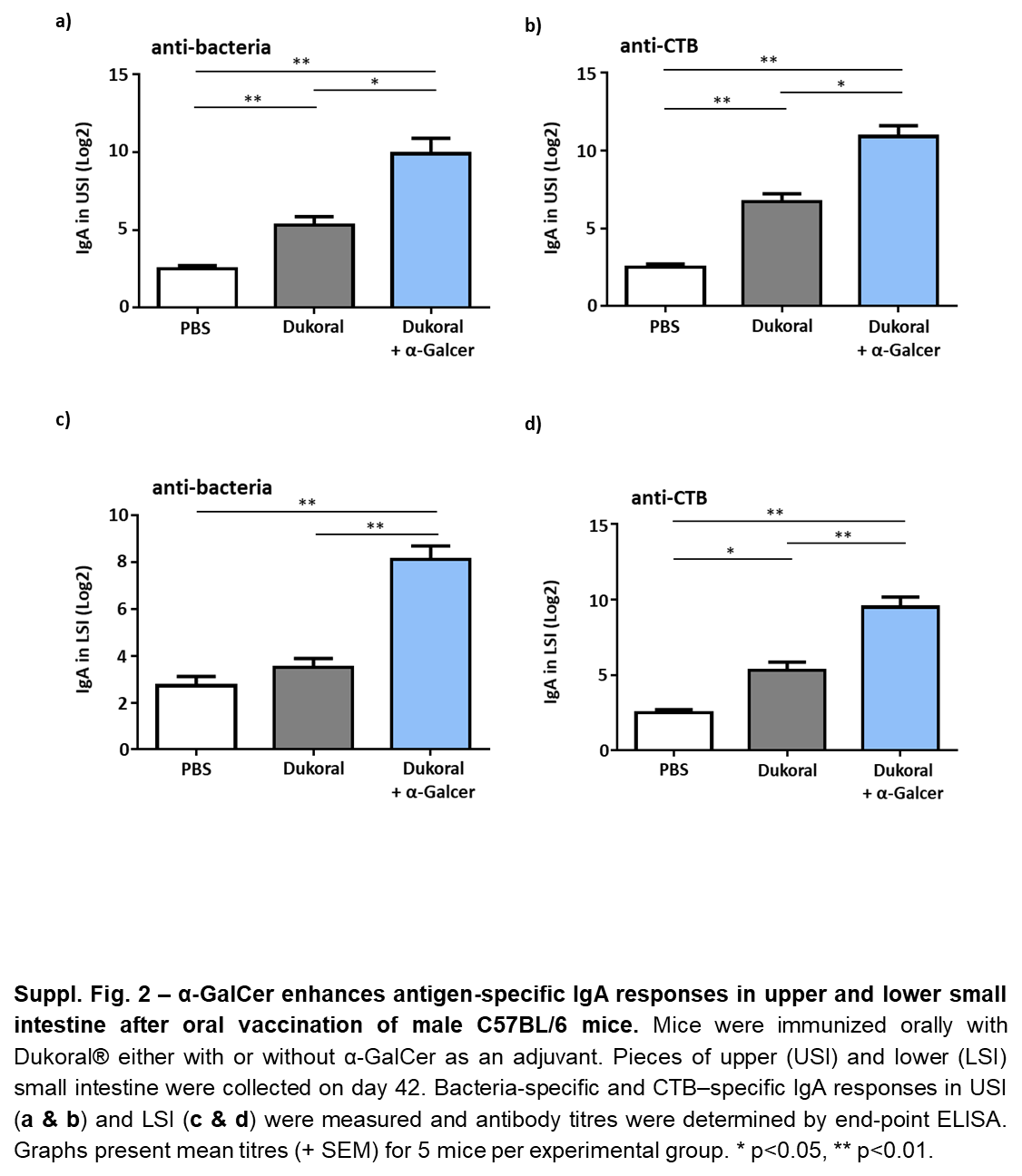


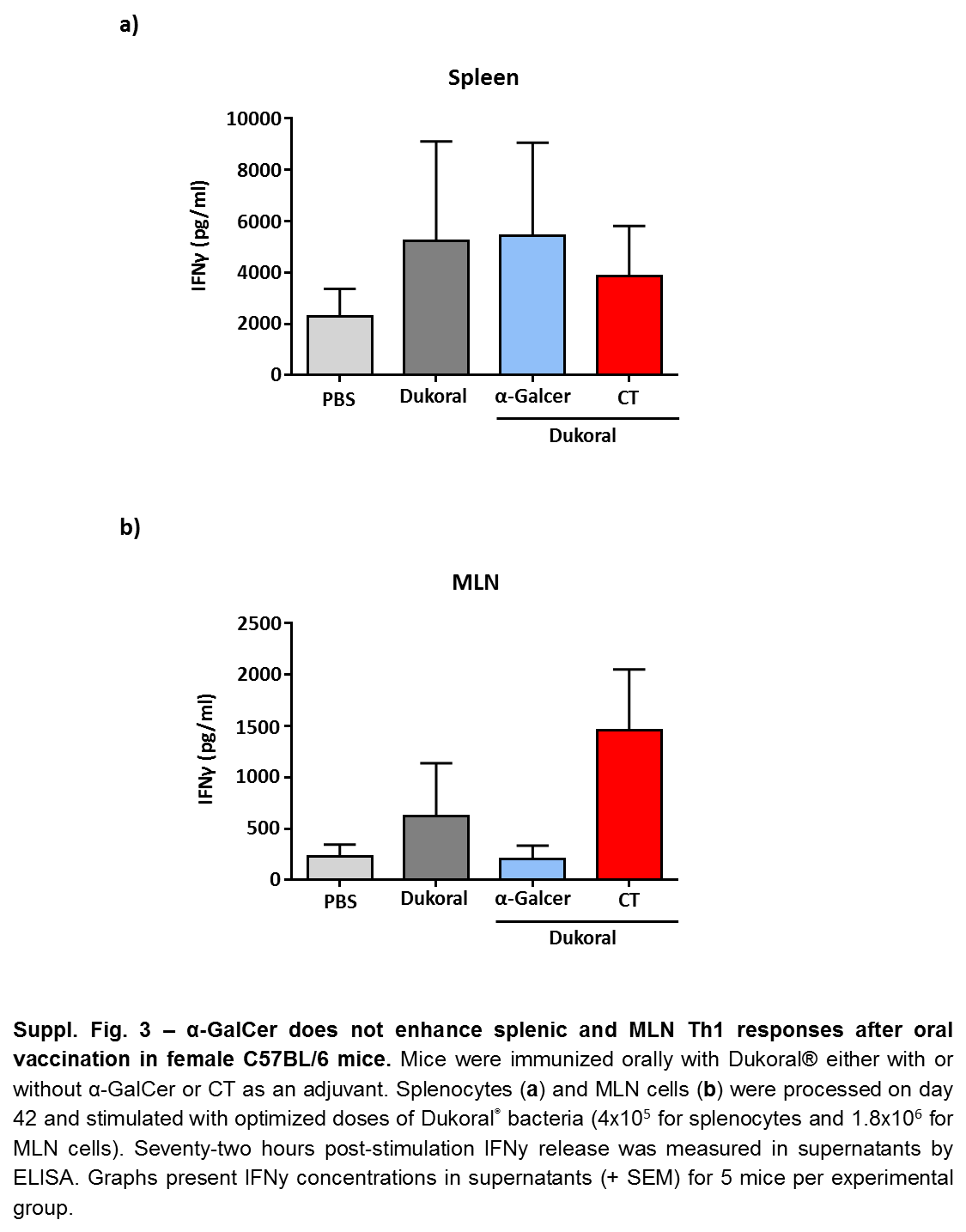


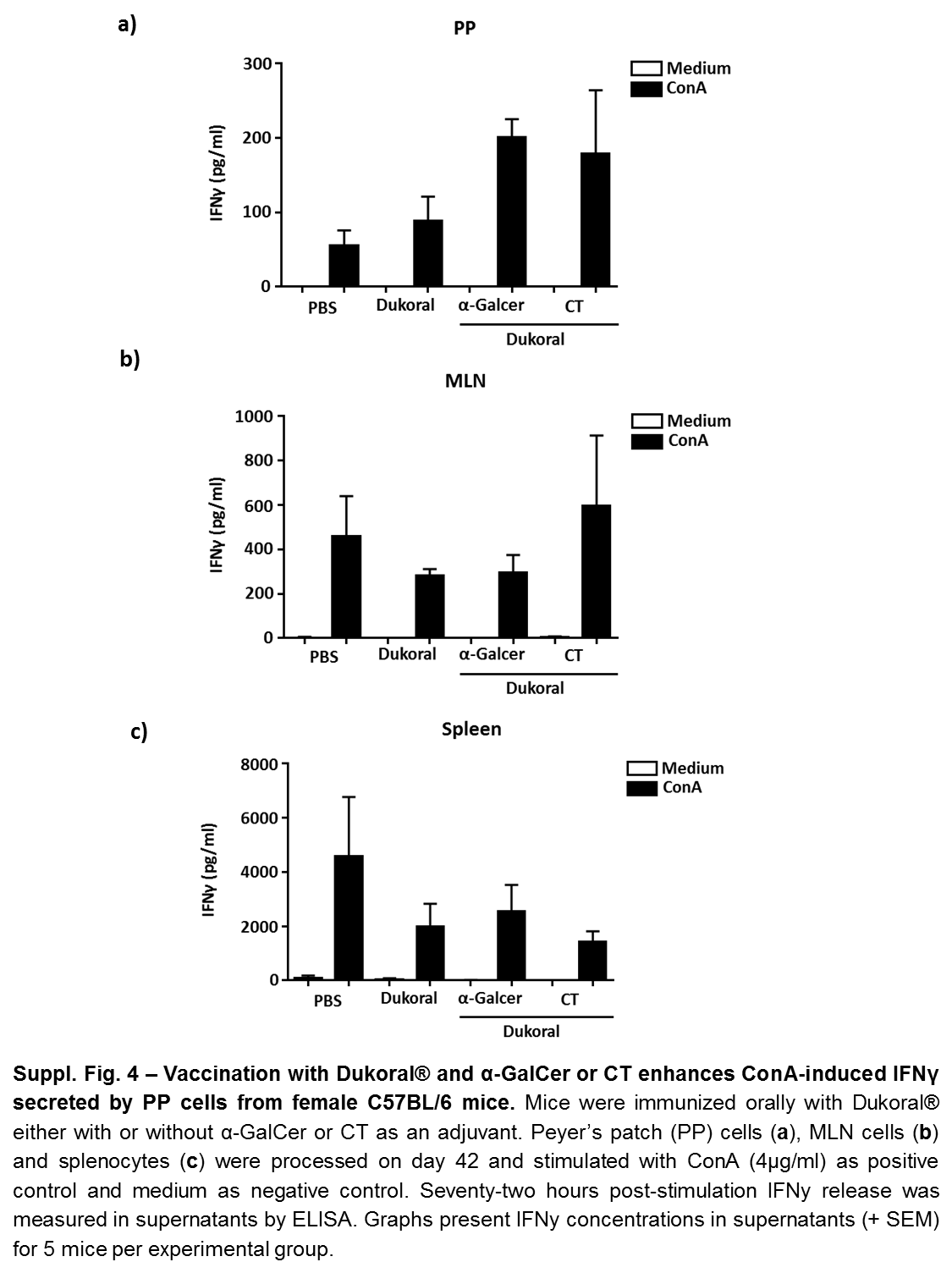


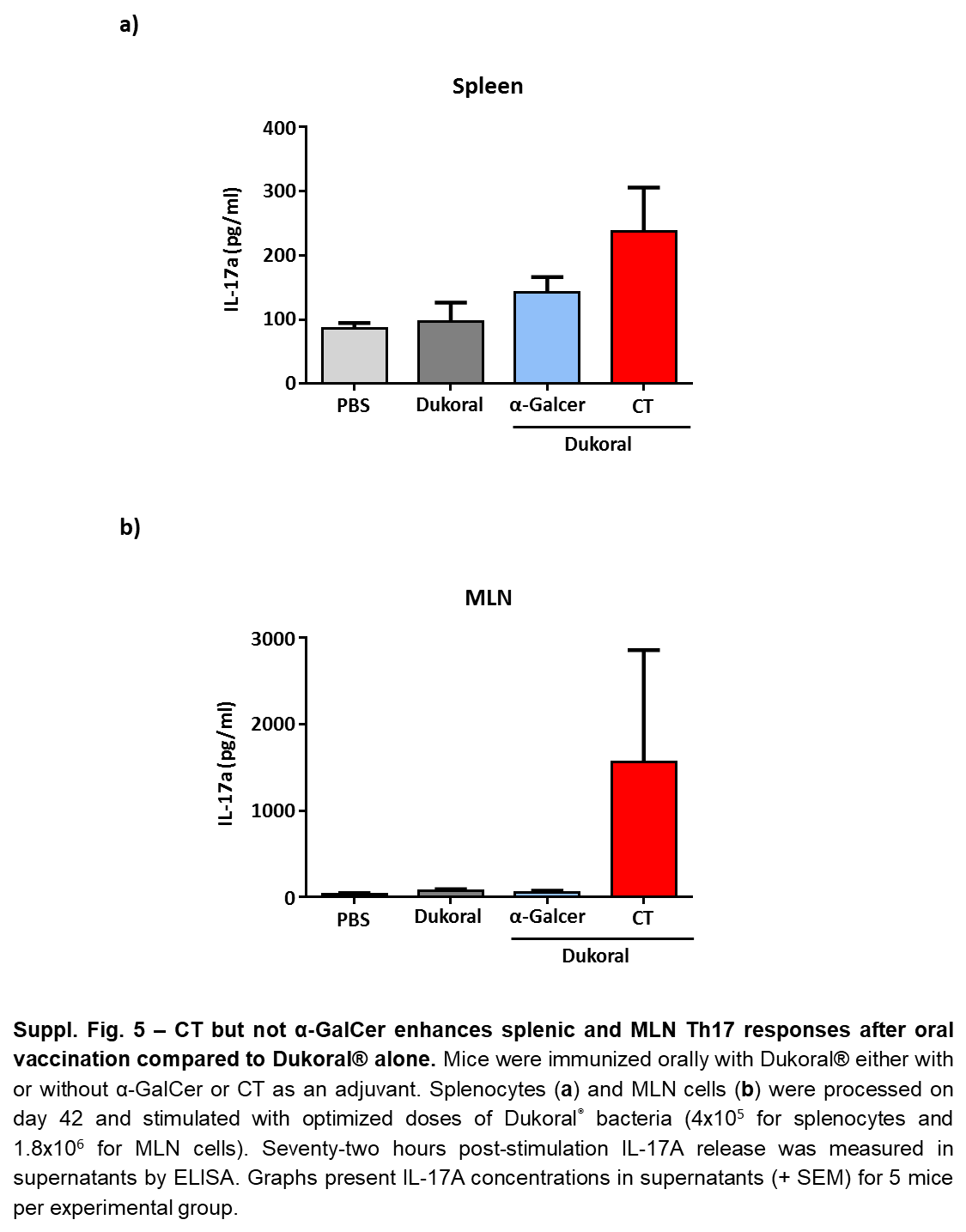


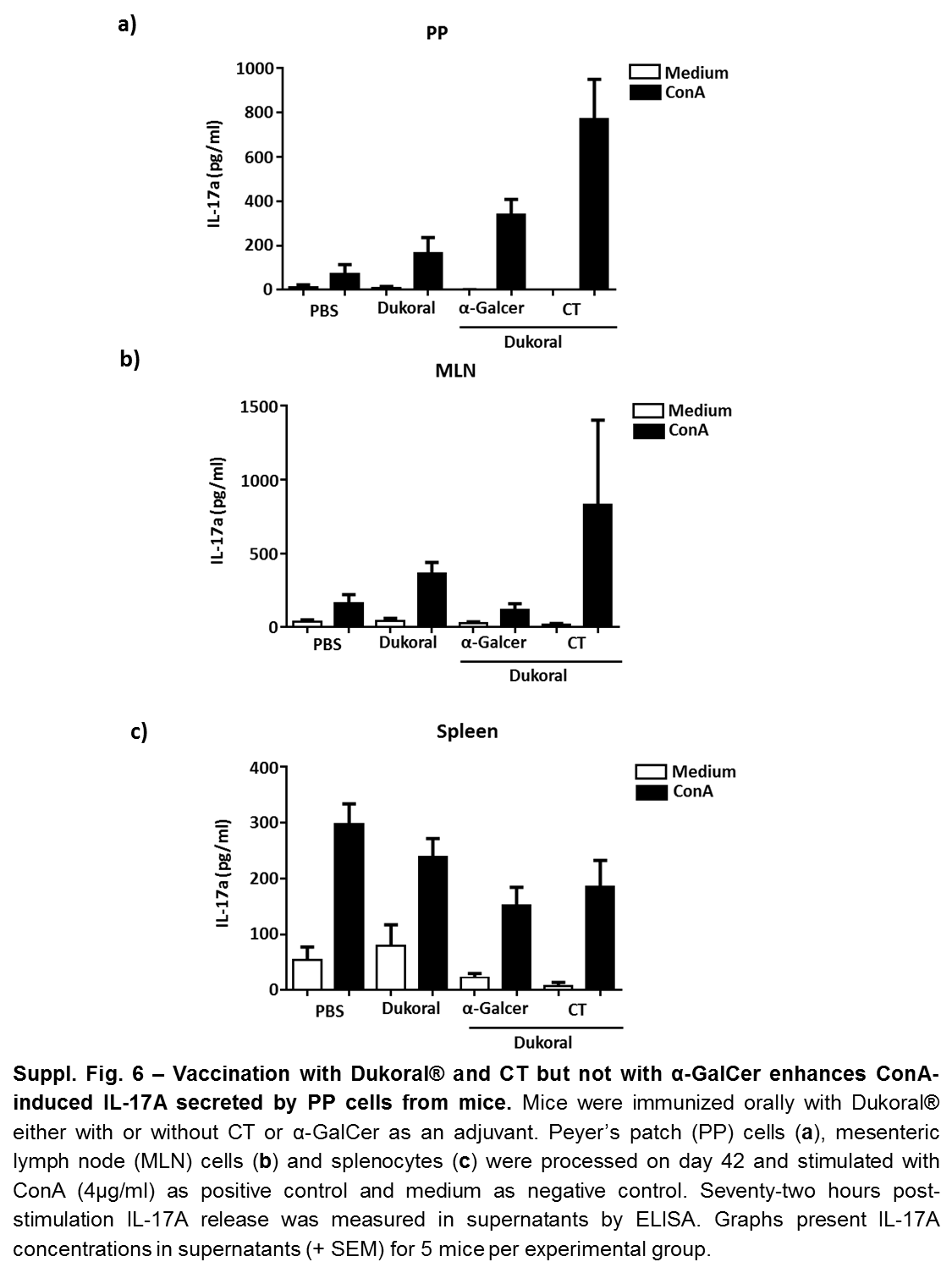


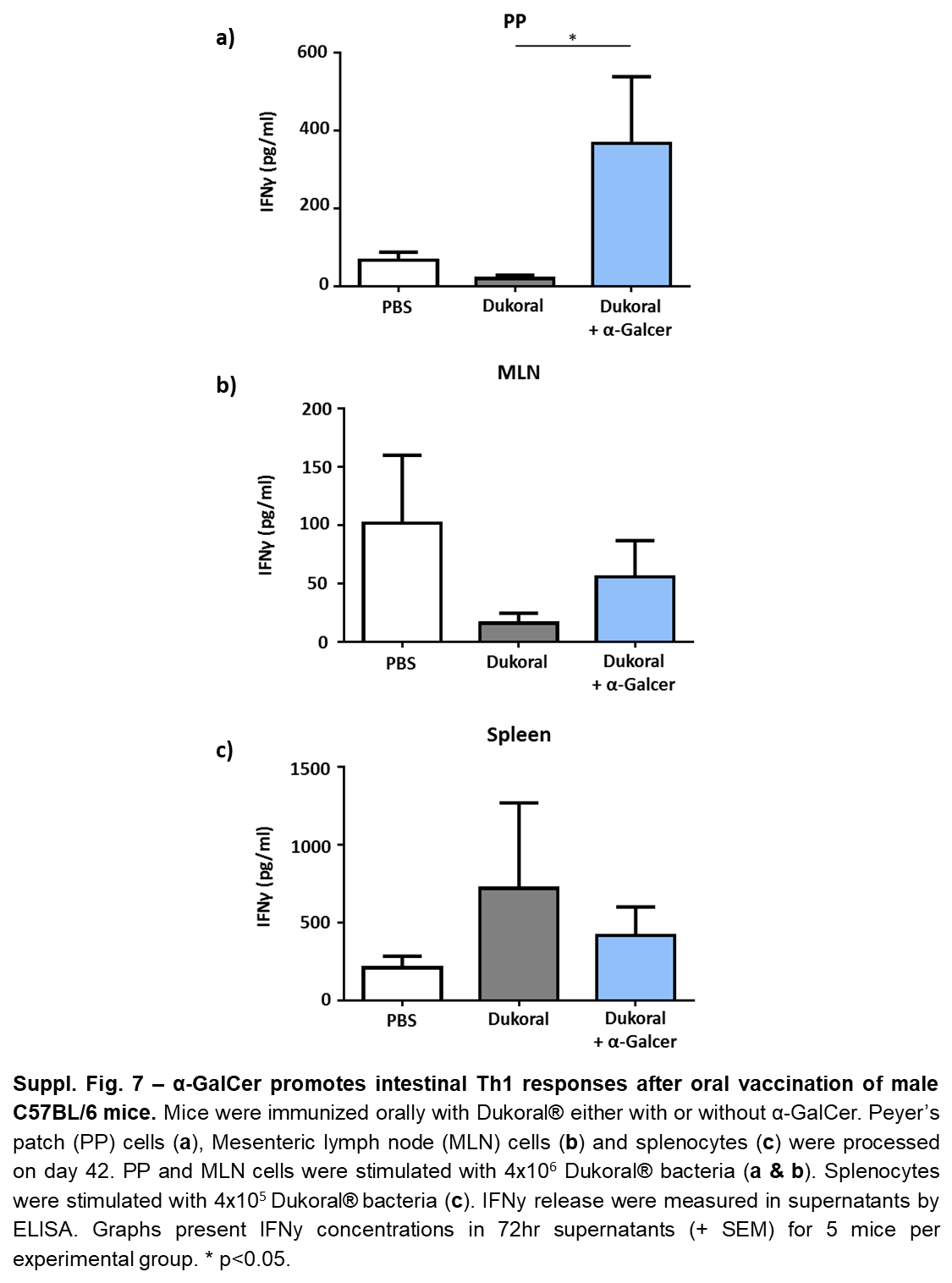


**
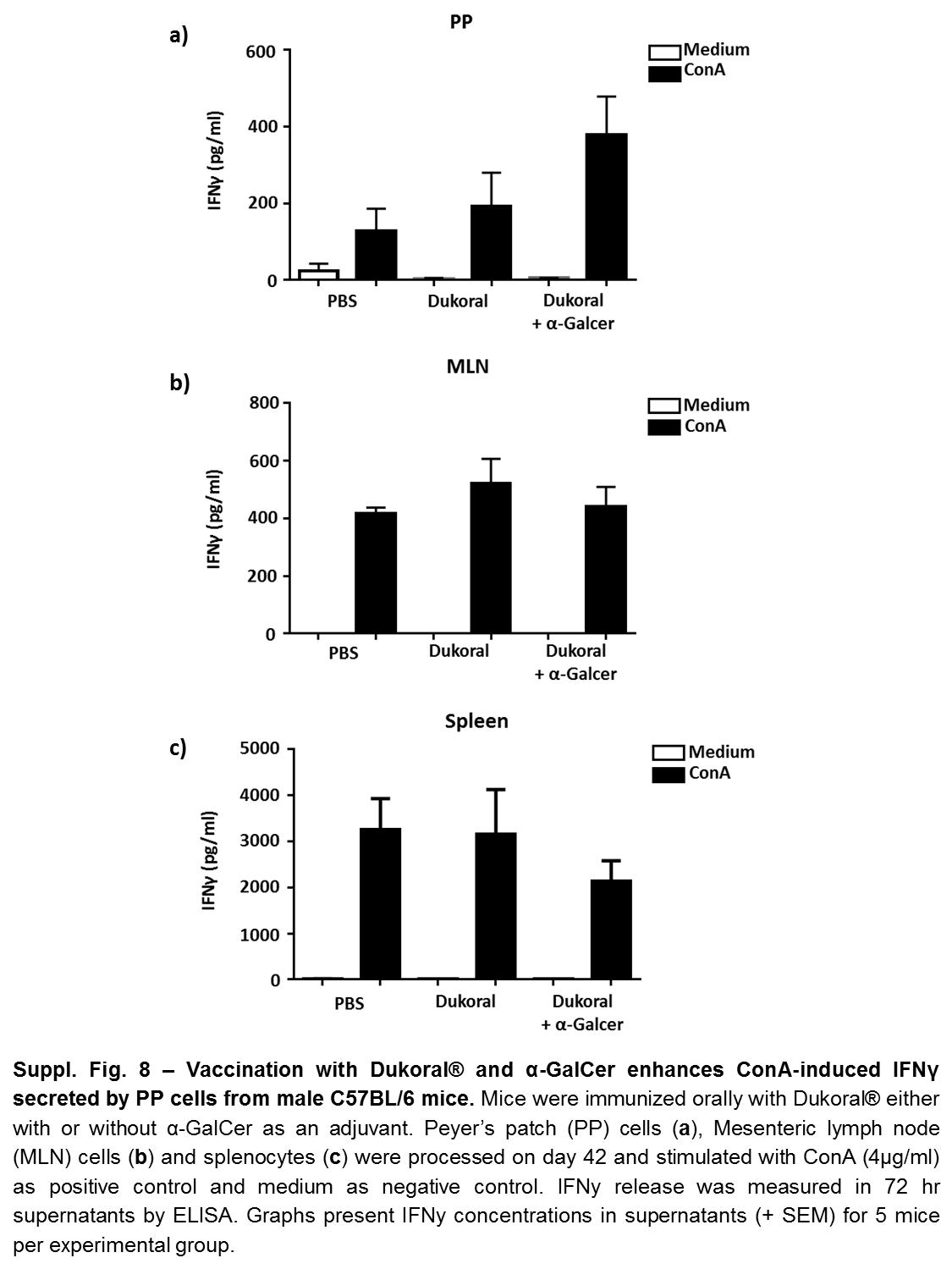
**


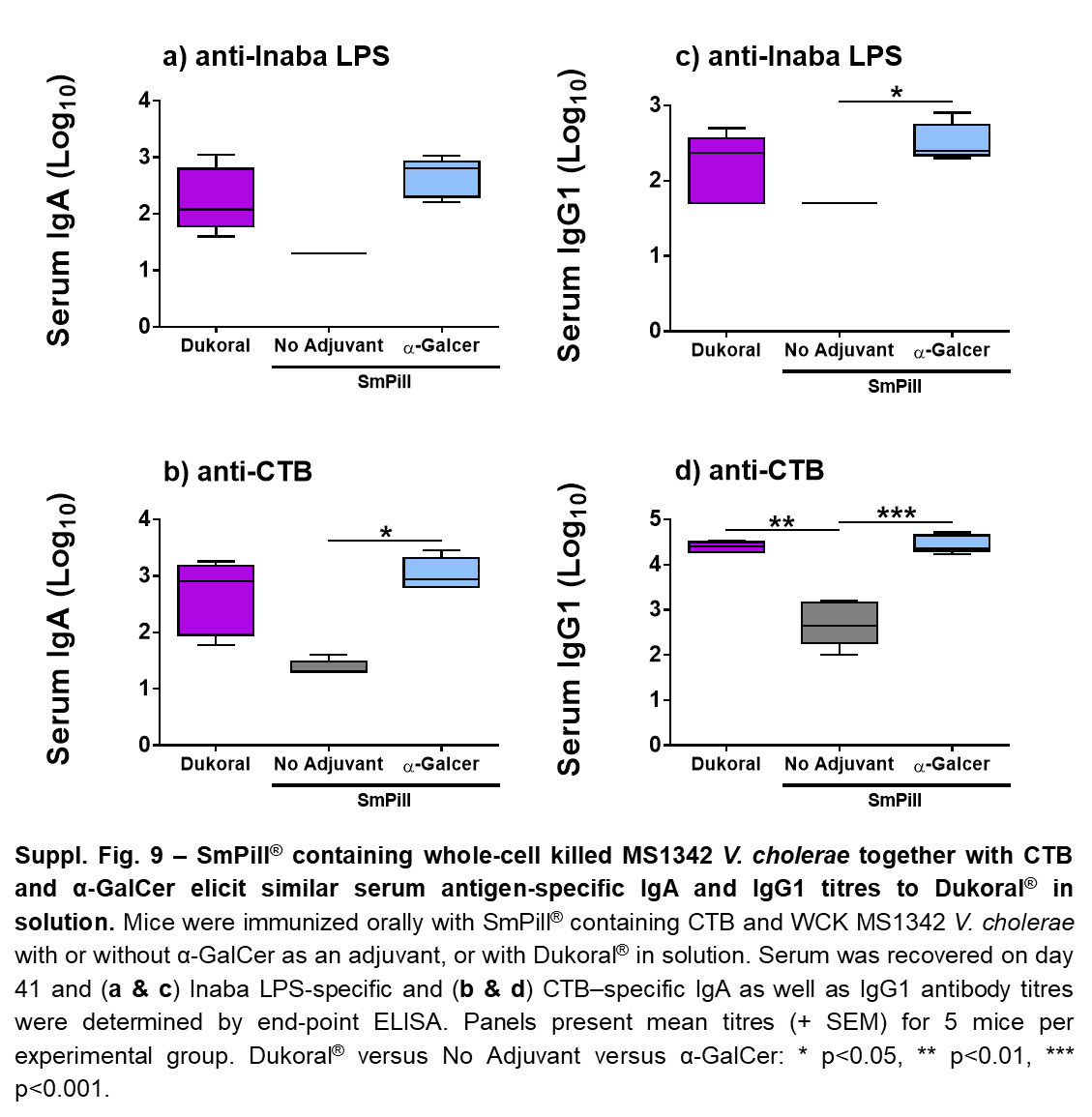

Supplement: Supplementary file 1 — Supplementary Material [file 41385_2019_159_MOESM1_ESM.doc]
